# Evolution of the IRF Family in Salmonids

**DOI:** 10.3390/genes12020238

**Published:** 2021-02-08

**Authors:** Thomas C. Clark, Pierre Boudinot, Bertrand Collet

**Affiliations:** VIM, UVSQ, INRAE, Université Paris-Saclay, 78350 Jouy-En-Josas, France; thomas-campbell.clark@inrae.fr (T.C.C.); Bertrand.Collet@inrae.fr (B.C.)

**Keywords:** teleost fish, type I interferon, paralogues, WGD, poly I:C, *Vibrio*, bacterin

## Abstract

Interferon regulatory factors (IRFs) as a family, are major regulators of the innate antiviral response in vertebrates principally involved in regulating the expression of interferons (IFNs) and interferon-stimulated genes (ISGs). To date, nine IRFs have been identified in mammals with a 10th member also found in several avian and fish species. Through genome mining and phylogenetic analysis, we identified and characterised 23 *irf* genes in 6 salmonid species. This larger repertoire of IRF in salmonids results from two additional whole-genome duplications which occurred in early teleosts and salmonids, respectively. Synteny analysis was then used to identify and confirm which paralogues belonged to each subgroup and a new nomenclature was assigned to the salmonid IRFs. Furthermore, we present a full set of Real-Time PCR primers for all rainbow trout IRFs, confirmed by sequencing to ensure paralogue specificity. RT PCR was then used to examine the response of all trout *irf* genes in vivo, following *Vibrio anguillarum* and poly I:C stimulation, indicating potential functional divergence between paralogues. Overall, this study presents a comprehensive overview of the IRF family in salmonids and highlights some novel roles for the salmonid-specific IRFs in immunity.

## 1. Introduction

Interferon regulatory factors (IRFs) are an ancient family of transcription factors present in all main contemporary groups of metazoans from sponges to mammals, with common ancestors dating back to 600 million years ago [1]. All IRF members are structurally similar, with each possessing a highly conserved N-terminal DNA binding domain (DBD) of around 115 amino acids characterised by 5 tryptophan rich repeats [2]. The DBD forms a helix-turn-helix structure allowing IRF proteins to recognise and bind to a DNA motif known as interferon (IFN)-stimulated response element (ISRE) [3]. The C-terminal region of these proteins is more variable, but in general, contains a nuclear export sequence, an autoinhibitory region and an IRF association domain (IAD) responsible for interacting with other IRFs and other transcription factors [2]. Two types of IAD have been identified, with IAD1 being present in all IRFs apart from IRF1 and IRF2 which instead contain the IAD2 [4]. IRF family members can both homo- or hetero-dimerise forming either transcriptionally active or repressive complexes [5]. Protein interactions within the IAD and other transcription factors likely determine whether the resulting complex acts as a transcriptional repressor or activator. The IRF family members can be grouped functionally by whether they are an activator (IRF1, IRF3, IRF5, IRF9 and IRF10), a repressor (IRF8), or whether they are multifunctional and can both repress and activate gene transcription (IRF2, IRF4 and IRF7) [6]. However, generally within the family, IRF1, IRF3, IRF5 and IRF7 function as positive mediators of the hosts IFN response whereas IRF2 and IRF4 act as repressors [4,7].

The IRF family members are major regulators of the innate antiviral response in vertebrates principally involved in transcriptional induction of interferons (IFNs) and interferon-stimulated genes (ISGs) [8]. Additionally, IRFs are known to be involved in metabolism control [9] and have extensive roles within immune cell development and maturation, reviewed in [10,11,12]. Viral infections are detected by animal cells through the recognition of virus-associated molecular patterns (PAMPs) via double stranded RNA by pattern recognition receptors (PRRs) such as members of the toll-like receptors (TLRs) family and retinoic acid-inducible gene I (RIG-I)-like receptors (RLRs) [13,14]. In vertebrates, these receptors activate convergent signalling pathways involving in particular IRF1, IRF3 and IRF7, and leading to the induction of specialized cytokines, the type I IFNs. Type I IFNs are secreted and have autocrine and paracrine actions after binding to their cognate IFN membrane-bound receptors. In mammals, type I IFN signalling depends on this interaction with the heterodimeric receptor complex comprised of IFNAR1 and IFNAR2 belonging to the class II cytokine receptor family [15,16]. These receptors are known as the cytokine receptor family B (CRFB) in fish, with CRFB1, 2 and CRFB5 being homologous to mammalian IFNAR2 and IFNAR1 [17]. Activation of type I IFN receptors induces a signalling cascade initiated by the phosphorylation of JAK1 and TYK2 kinases (reviewed in [18,19]), and leading to the association of IRF9 with the STAT1 and STAT2 molecules to form the interferon-stimulated gene factor 3 (ISGF-3) [20,21,22]. After translocation into the nucleus, ISGF-3 binds to specific motifs located in the promoters of more than a hundred IFN-stimulated genes (ISGs) with effector and regulatory functions [23]. This complex system is highly regulated and can produce diverse responses depending on viral detection and subversion mechanisms, as well as cell type and activation state. Members of the IRF family play crucial roles at multiple levels of the IFN signalling, and in its regulation.

To date, nine IRFs have been identified in mammals (1–9) with a 10th member in several avian and fish species [24]. Phylogenetic analysis of the vertebrate IRF family, reveals members can be subdivided into four subgroups reflecting their evolutionary history: IRF1-G (IRF1, IRF2), IRF3-G (IRF3, IRF7), IRF4-G (IRF4, IRF8, IRF9, IRF10) and IRF5-G (IRF5, IRF6) [1]. The repertoire of *irf* genes is larger in bony fishes, likely stemming from the whole-genome duplications (WGD) this group was subjected to: the number of IRF present in the last common ancestor of teleosts and tetrapods (LCATT) was doubled by the teleost-specific WGD (tsWGD) that occurred at the root of this lineage about ~300 million years (Myrs ago), then increased again by additional WGD events in particular groups such as salmonids, and carps [25]. Many fish irf paralogs have been described, especially in salmonids such as *irf7a/b* [26] and *irf10a/b* [27].

Based on high-quality genome assemblies, we present a comprehensive characterisation of 23 *irf* genes within 6 salmonid species (*Oncorhynchus mykiss*, *Oncorhynchus nerka*, *Oncorhynchus tshawytscha*, *Oncorhynchus kisutch*, *Salmo salar* and *Salmo trutta*) with a focal point on rainbow trout (*O. mykiss*). Salmonids underwent a fourth round of genome duplication 88–103 Myrs ago termed the salmonid-specific whole-genome duplication (ssWGD) [25,28], leading to a large number of *irf* genes. We investigated their phylogenetic relationships and syntenies to understand their origin, i.e., whether paralogs were produced by the tsWGD or by the ssWGD. We also present additional evidence for the existence of an 11th member of fish IRF, often referred to as IRF1a, and an updated nomenclature of fish IRF. Finally, we have designed a paralogue-specific primer set for all IRFs whose expression profiles were examined in rainbow trout spleen tissue after 24 h stimulation with viral and bacterial PAMPs. Our data offers a novel insight into the evolution of the IRF family in salmonids which is coupled with functional data that highlights potential functional divergence within the salmonid-specific IRFs.

## 2. Materials and Methods

### 2.1. Phylogenetic and Gene Synteny Analysis

Protein sequences of IRF family genes for *O. mykiss* (GCF_002163495.1), *O. nerka* (GCF_006149115.1), *O. tshawytscha* (GCF_002872995.1), *O. kisutch* (GCF_002021735.2), *S. salar* (GCF_000233375.1) and *S. trutta* (GCF_901001165.1) were originally obtained from BLASTp searches with known zebrafish and human orthologues of IRFs 1–10 as the query. Protein sequences of all IRFs for all the salmonids, human, chicken, zebrafish, northern pike and spotted gar were retrieved from NCBI or Ensembl if it could not be located on NCBI (for full list of protein sequences, gene IDs and accession numbers, see Table S1). In situations where several isoforms were found, the longest was chosen. Protein sequences were aligned using the ClustalW method in the MEGA-X software [29]. The phylogenetic tree was then constructed using the neighbour-joining and ML methods in MEGA X (ML trees can be viewed in Figures S1 and S2), corrected using the Poisson model, and bootstrapped 2000 times. A second phylogenetic tree of only IRF1 and IRF2 subfamilies with IRF3 as an outgroup was also constructed for more species with the same parameters. Gene synteny analysis was carried out for all *irf* genes between salmonid and other relevant genomes. To determine the genomic neighbourhood around candidate genes and the conservation of gene order across species, genes were visually examined in NCBI’s genomic region browser.

### 2.2. IRF Primer Design

Due to the duplicated nature of salmonid genomes, care was taken to design paralogue-specific primers. Nucleotide transcript sequences from all *irf* genes were aligned by their IRF family (i.e., IRF1, IRF2) in Clustal Omega in order to identify divergent regions suitable for specific primer design. Primers were designed to span 100–200 bps where possible and the annealing temperature was identified using OligoCalc. Primer specificity was confirmed through sequencing of the reverse and forward strands of PCR products amplified from each experimental condition shown in Section 3.5, i.e., 1 PBS, Poly I:C and *Vibrio* extract.

### 2.3. Animal Work

Adult rainbow trout were raised in the fish facilities of Institut National de la Recherche en Agriculture et environnement (INRAE, Jouy en Josas, France). Fish (*n* = 4) were injected intraperitoneally (IP) with 100 µL of either PBS, poly I:C (Sigma catalogue# P1530; 5 µg per g of fish) or *V. anguillarum* extract (diluted 1/10). To prepare extracts, *V. anguillarum* strain PO382 was grown in tryptic soy broth medium to OD600 (optical density at 600 nm) 1.5. Bacterial pellet (from 10 mL of full-grown culture) was resuspended in NaCl (9 g/L), and the suspension was washed four times in NaCl (9 g/L) and resuspended in 1 mL of the same isotonic solution and incubated 0.5 min at 100 °C to kill bacteria, as described in [30]. Fish were then kept for 24 h within 1 tank (300 L) supplied with recirculating dechlorinated water with a flow rate of 1000 L/h, temperature of 10 °C, and a photoperiod of 10:14 light:dark. A computerised control system was used to monitor pH, ammonia concentration and oxygen levels over the duration of the stimulation. Fish were sacrificed by overexposure to benzocaine. Spleen tissue (100–200 mg) was extracted from each fish and stored in RNA later at 4 °C overnight before long term storage at −80 °C.

### 2.4. RNA Extraction and Reverse Transcription

Total RNA was extracted from 100 mg of tissue homogenised with ceramic beads in a FastPrep-24 5 G tissuelyser in 1 mL of Trizol following the manufacturer’s instructions. Concentration and purity of RNA was estimated using a Nanodrop 2000 C Spectrophotometer. First strand cDNA was synthesised from 1 μg RNA using a Biorad iScript advanced cDNA kit with an integrated genomic DNA elimination step. First strand cDNA samples were diluted 20-fold (working stock) with RNase/DNase free water (Sigma, St. Quentin Fallavier, France) and stored at −20 °C.

### 2.5. Quantitative Real-Time PCR

Real-time PCR (qPCR) analyses were performed with an Eppendorf Realplex2 Mastercycler. All assays were carried out in 15 μL reactions on 96-well plates in duplicates. Reaction mixes each contained 5 μL cDNA, 15 μL Biorad iTaq Universal SYBR Green Supermix and 5 μL of reverse and forward primer (250 nm each). PCR cycling conditions were 1 cycle of 95 °C for 3 min, followed by 40 cycles of 95 °C for 10 s then between 55 and 62 °C for 20 s (two-step PCR). Melting curve analysis (thermal gradient from 55 to 95 °C) was then used to confirm the amplification of a single product. Each plate also included “no template” negative controls in duplicate (cDNA replaced with water). Efficiency was calculated for each primer from a serial dilution PCR ran alongside. Target gene expression was normalised to β actin and then relative expression levels calculated. Full primer list, sequence and annealing temperatures can be viewed below in Section 3.3.

### 2.6. Statistical Analysis

Statistical analysis of qPCR data was performed in R (v4.0.4) using expression data calculated from ∆∆Ct method. A linear model (lm) was first made in R and the diagnostic plots (qq plot and residuals versus fitted values) were assessed in order to ensure both normality and equal variance. If data met the assumptions, the one-way ANOVA results from R’s linear model function could then be interpreted and a post-hoc Tukey test performed.

## 3. Results

### 3.1. Phylogenetic Analysis of Salmonid IRF Family

**Figure 1:**
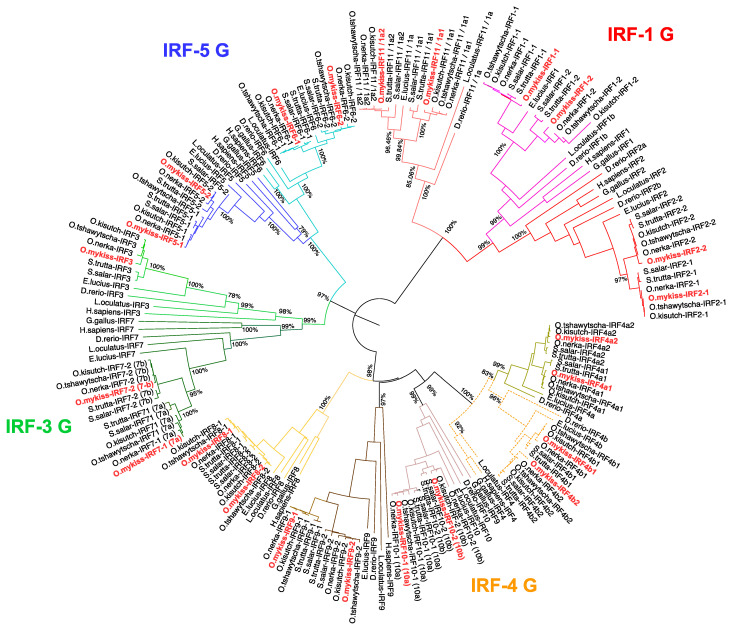
Phylogenetic tree showing the evolutionary relationship of interferon regulatory factor (IRF) transcription factors in salmonids. IRF protein sequences were aligned using ClustalW in the MEGA-X software. Following alignment, the phylogenetic tree was constructed using the neighbour-joining method in MEGA X and corrected using the Poisson model. The branch support values were gained by non-parametric bootstrapping (2000 replicates). Branches have been coloured to represent evolutionary groups; rainbow trout sequences have also been coloured in red for ease of visibility. The scale bar represents the calculated evolutionary distance. Genbank accession numbers for all species can be viewed in Table S1.

**Figure 2:**
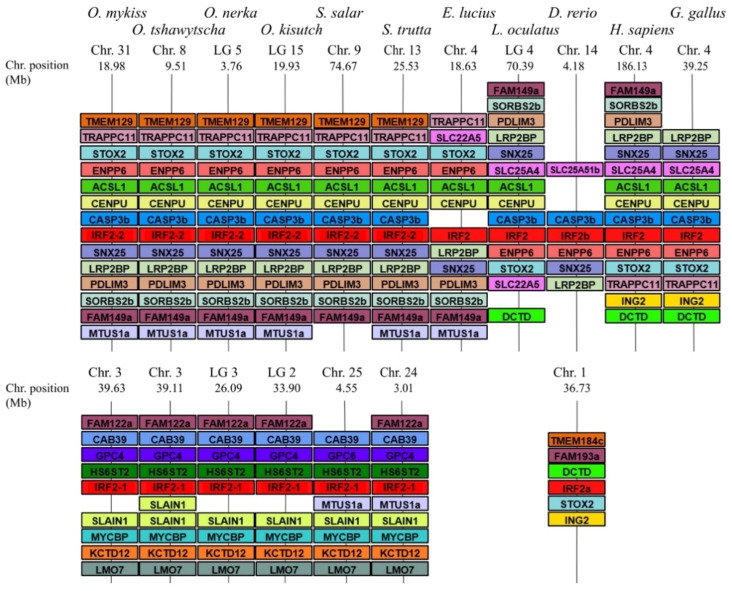
IRF2 gene synteny in salmonids. The syntenically conserved gene blocks are shown in matching colours. Gene synteny was compiled from up- and down-stream locations relative to each species IRF2 taken from NCBI’s genome browser. Species names are displayed at the top of the figure, IRF gene accession numbers are on Table S1, chromosome number and range (position) are shown above and below each species gene synteny.

A total of 23 IRF family members in *O. mykiss*, *O. kisutch*, *O. nerka*, *S. salar* and *S. trutta* were identified from genome data on NCBI, through BLASTp searches with human and zebrafish orthologues as the query. Only 21 *irf* genes could be found from *O. tshawytscha* (one *irf5* and one *irf10* were missing). Care was also taken to exclude *irf*-like genes which share similar domains to IRFs, such as the sex-determining gene (*sdY*) in rainbow trout which contains an IAD domain similar to IRF9 but no typical DBD [31]. Phylogenetic analysis of sequences from salmonids, human (*Homo sapiens*), chicken (*Gallus gallus*), pike (*Esox Lucius*), zebrafish (*Danio rerio*) and spotted gar (*Lepisosteus oculatus*) shows that they can be grouped into four main groups: IRF-1G (*irf1b*, *1a/11* and *irf2*), IRF-3G *(irf3* and *7*), IRF-4G (*irf4*, *8*, *9* and *10*) and IRF-5G (*irf5* and *6*) (Figure 1), as previously proposed [6,32,33]. All salmonid *irf* genes were orthologous to known human IRF and all but *irf3* have retained duplicated copies. In most cases (i.e., *irf5*, *6*, *7*, *8*, *9*, and *10*), the presence of two salmonid paralogues on distinct chromosomes and the branching of northern pike as a sister group with a unique copy confirms these genes to be products from the ssWGD. The situation was different for *irf2*, with two copies both in zebrafish and in salmonids, suggesting an older origin. *irf1* and *irf4* were revealed to have also duplicated copies in the other fish species analysed, suggesting they originated from the tsWGD. However, the structure of the IRF-1G subtree revealed a potential issue with the existing nomenclature due to the clustering of *irf1b* and *irf2* leaving *irf1a* as a well-supported outgroup, both in neighbour-joining and maximum likelihood phylogenetic analyses. The presence of both *irf1a* and *irf1b* in the spotted gar, a species belonging to a fish lineage that diverged before the tsWGD, indicates that these two genes did not originate from this WGD event. This is unlike the situation with *irf4*, where only other teleosts have *irf4a* and *irf4b* while the spotted gar only has one copy indicating *irf4a* and *irf4b* are products from the tsWGD. Gene synteny analysis was then carried out for all *irf* genes using the genomic region browser in NCBI in order to determine whether the chromosomic environment of *irf* genes across species supports relationships inferred from phylogenetic trees; *irf2* was used as a representative example (Figure 2), the rest of the *irf* gene synteny can be viewed in Table S2. Detailed analysis of the genomic environment of each copy supported the relationships inferred from phylogenetic analyses for family members belonging to groups 2–4.

### 3.2. Comparative Phylogenetic and Synteny Analysis on the Case of Group 1 Salmonid Irfs

**Figure 3:**
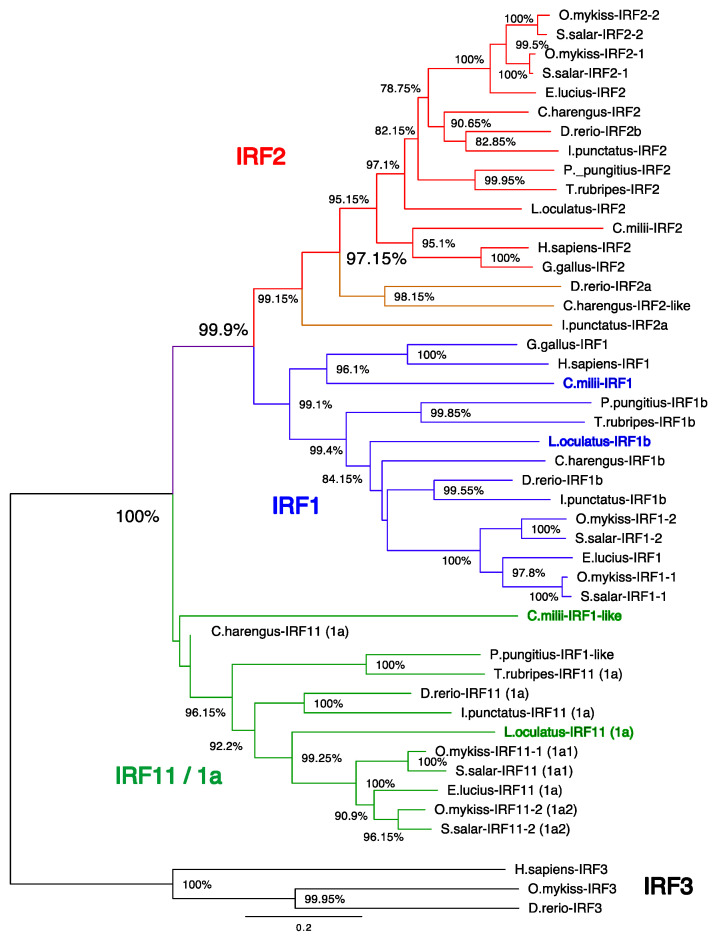
Phylogenetic tree showing the evolutionary relationship of IRF1 and IRF2 transcription factors in vertebrates with IRF3 used as an outgroup. IRF protein sequences were aligned using ClustalW in the MEGA X software. Following alignment, the phylogenetic tree was constructed using the neighbour-joining method in MEGA X and corrected using the Poisson model. The branch support values were gained by non-parametric bootstrapping (2000 replicates). The scale bar represents the calculated evolutionary distance. Genbank accession numbers for all species can be viewed in Table S1.

**Figure 4:**
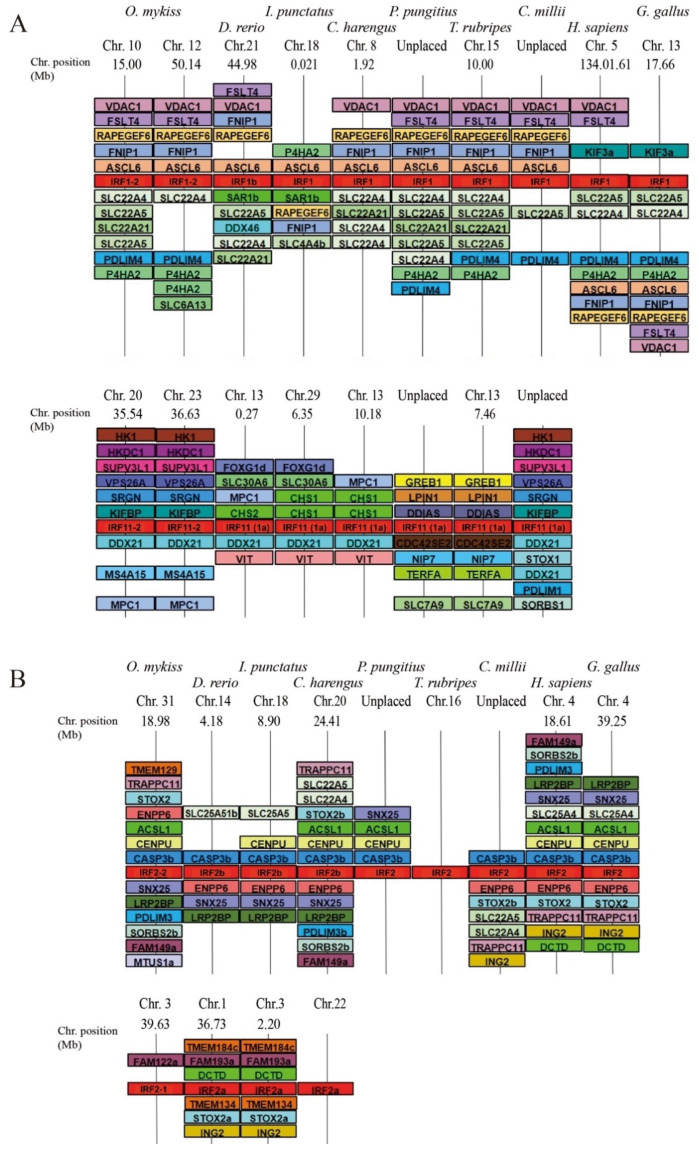
IRF1(**A**) and IRF2 (**B**) comparative gene synteny in vertebrates. The syntenically conserved gene blocks are shown in matching colours. Gene synteny was compiled from up and downstream locations relative to each species IRF taken from NCBI’s genome browser. Species names are displayed at the top of the figure, *irf* gene accession numbers are on Table S1, chromosome number and range (position) shown above and below each species gene synteny.

To further investigate the evolutionary relationship of *irf1* and *irf2* within fish species another phylogenetic (Figure 3) and synteny analysis (Figure 4) was performed with added species from more taxonomic groups: elephant shark (*Callorhinchus milii*), herring (*Clupea harengus*), channel catfish (*Ictalurus punctatus*), stickleback (*Pungitius pungitius*) and pufferfish (*Takifugu rubripes*).

As with the initial analysis, *irf1a/11* appears as a clear outgroup to *irf1b/1* and *irf2* (Figure 3). The presence of *irf1a/11* in cartilaginous fish (elephant shark) and spotted gar further confirms this IRF as its own sub-family in fish that was likely an ancestral IRF lost in tetrapods. The genomic neighbourhood of tetrapod *irf1* and fish *irf1b* was found to be highly similar, while the genomic neighbourhood of fish *irf1a* was divergent (Figure 4). The only possible similarity between *irf1/1b* in tetrapods and the fish-specific *irf1a/irf11* was the presence of a gene belonging to the kinesin family: the kinesin-like *kif3a* was located close to *H. sapiens* and *G. gallus irf1*, and the *kifbp* (Kinesin family binding protein) was observed close to teleost *irf1a* (Figure 4).

In contrast, the genomic neighbourhood of the fish-specific *irf1a/11* was not conserved (Figure 4). Three separate groupings of synteny can be observed: salmonid *irf1a1* (or *irf11-1*) and *irf1a2* (or *irf11-2*) were highly similar to elephant shark, likely representing the primordial configuration (Figure 4, in which rainbow trout was used as a representative of the salmonids). Zebrafish, catfish and herring *irf1a* regions all shared a high degree of similarity amongst each other with a few shared genes (*ddx*, *chs* and *mpc1*) that are also present in salmonids. Stickleback and pufferfish *irf1a* regions are very similar to each other, but they had no marker in common with the other species analysed (Figure 4).

In the case of *irf2* (Figure 3), two paralogs were found in additional fish species like the channel catfish and herring, while most teleosts apparently retained only one copy. This copy of *irf2* (*irf2-2* in salmonids) is well conserved throughout the fish species and is orthologous to tetrapod *irf2*, as reflected in the phylogenetic tree.

There is significant conservation of the genomic context between species, although a high degree of gene shuffling and variation has occurred among what is conserved. For example, the pufferfish *irf2* neighbourhood shared no similarity with any other species (Figure 4).

In contrast, the second copy (named *irf2-1* in salmonids and *irf2a* in zebrafish) which was only found within salmonids, zebrafish, catfish and herring, did not group as a consistent set in the phylogenetic tree (Figure 3). Salmonid *irf2-1* grouped with *irf2-2*, while in other species *irf2a* were more divergent. Zebrafish and catfish *irf2a* regions shared an identical gene order while both the rainbow trout and herring *irf2a* neighbourhoods shared almost no similarity with the other *irf2a*. The lack of similarities between the *irf2* copy from different species may suggest these genes have arisen from different/independent duplication events (not only WGD) or were subjected to further rearrangements.

Based on our genomic overview of salmonid *irf* genes, we then developed a comprehensive set of primers allowing specific amplification and quantification of mRNAs encoding each rainbow trout paralog (Table 1).

### 3.3. A Consistent Nomenclature for Salmonid IRF Family

The combined phylogenetic and synteny analysis led us to propose a consolidated and coherent nomenclature of salmonid *irf* genes similar to the one we recently reported for stat genes [37]. The letter a/b corresponding to the tsWGD (and to genes generally present in the zebrafish), and the subsequent number (1 or 2) identifying the copies produced by the ssWGD, based upon chromosome number in rainbow trout. For most of the salmonid IRFs here, this was the first time they have been characterised. However, in cases where salmonid paralogues have been characterised previously (i.e., *irf7a* and *irf7b*, *irf10a* and *irf10b*), we suggest that the “a” paralogues are now labelled as 1 and the “b” paralogues are labelled as 2, respecting the original characterisation. For example, *irf7a* and *irf7b* would now be called *irf7-1* and *irf7-2*, respectively, acknowledging them as products of the ssWGD. For the remaining results and discussion, we will now refer to the salmonid IRFs by their proposed name. The full list of proposed IRF family gene names and paralogue-specific primers is provided in Table 1 and Table S1.

### 3.4. Constitutive mRNA Expression Levels of the IRF Gene Family

**Figure 5:**
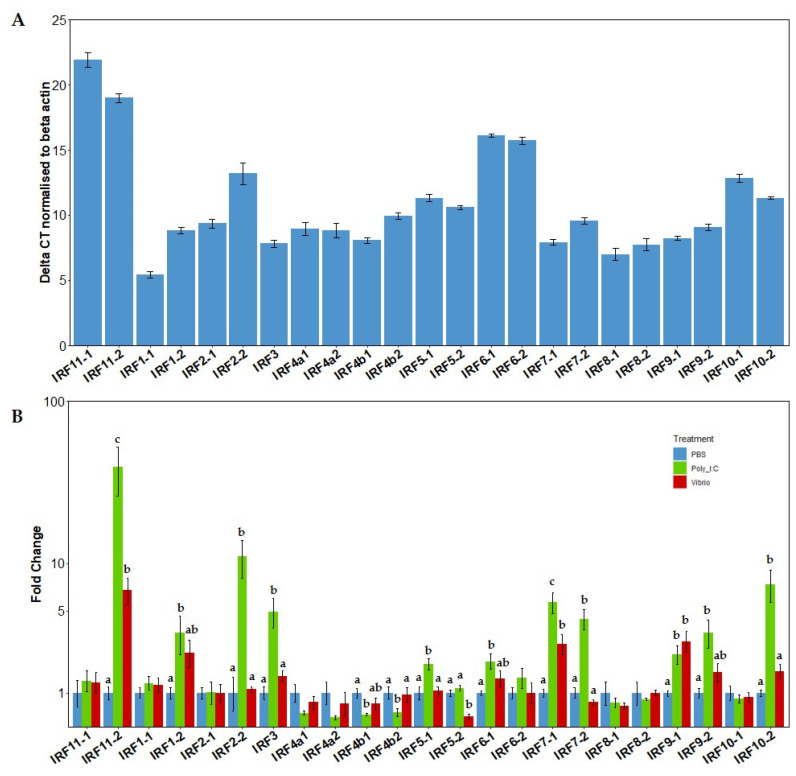
(**A**). Relative constitutive expression of IRFs in control (PBS) fish spleen tissue. Bars represent the delta CT (DCT) of the IRFs when normalised to the house keeping gene β actin (±SEM), *n* = 4. (**B**). Fold change expression of IRF family members in spleen tissue of rainbow trout. Fish were injected i.p. with either PBS, *V. anguillarum* extract or poly I:C for 24 h. Expression was normalised to the housekeeping gene β actin. A linear model was used for analysis of all genes. Bars represent fold change relative to the PBS control group (±SEM), *n* = 4. Results of the Tukey post hoc test are displayed above the bars. Bars which do not share a letter are significantly different.

The relative mRNA basal expression levels of the *irf* gene family repertoire were initially analysed within spleen tissue of rainbow trout from the control (PBS) group (Figure 5A). Gene expression of the *irf* were displayed as the delta Ct values after normalization with β actin (Figure 5A). Several members of the *irf* family (*irf 11-1*, *irf 11-2*, *irf 6-1* and *irf 6*-*2*) were very poorly expressed in the unstimulated spleen tissue. Following *irf1-1*, *irf8-1* was the second most expressed gene, which was then followed by *irf8-2* and then *irf3*, although constitutive expression of *irf8-2* was much more variable.

### 3.5. mRNA Expression Levels of the IRFs in Response to Poly I:C or Vibrio Extract

**Figure 6:**
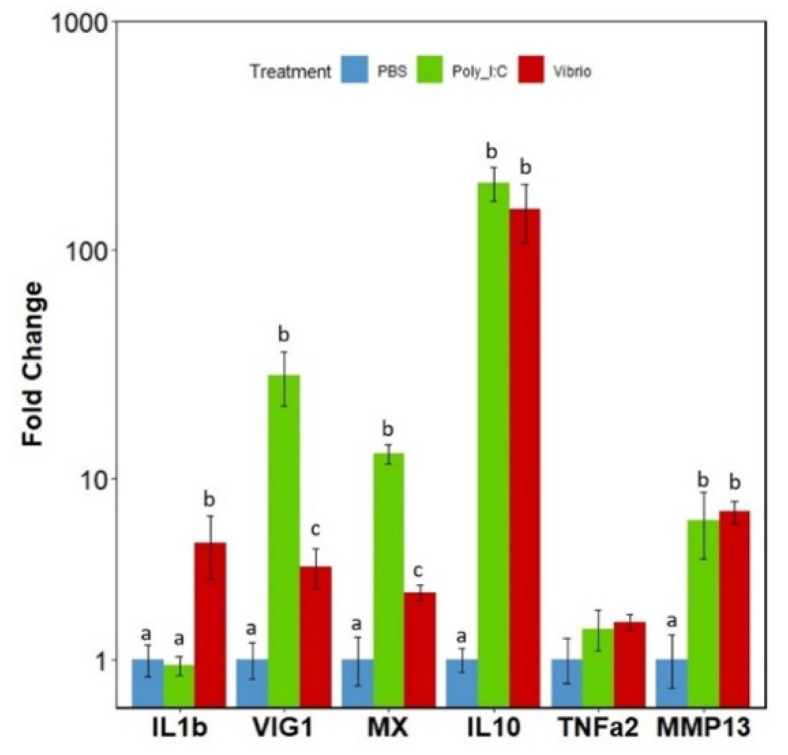
Fold change expression of inflammation marker genes: *interleukin 1 β* (IL1b), *viperin* (vig1/rsad2), *interferon-induced GTP-binding protein 3* (mx3), *interleukin 10* (il10) and *matrix metalloproteinase 13* (mmp13) in spleen tissue of rainbow trout. Fish were injected i.p. with either PBS, *V. anguillarum* extract or poly I:C for 24 h. Expression was normalised to the housekeeping gene β actin. A linear model was used for analysis of all genes. Bars represent fold change relative to the PBS control group (±SEM), *n* = 4. Results of the Tukey post hoc test are displayed above the bars. Bars which do not share a letter are significantly different.

Expression of the complete *irf* family gene repertoire was then examined in adult rainbow trout spleen tissue, following in vivo activation by intra-peritoneal injection of *V. anguillarum* extract or poly I:C to elicit either an antibacterial or antiviral response, respectively. Initially, to confirm fish were undergoing an inflammatory response to the stimulations the expression of several marker genes for viral and bacterial responses were examined: interleukin 1 β (*il1b*), viperin (*vig1/rsad2*), interferon-induced GTP-binding protein 3 (*mx3*), interleukin 10 (*il10*) and matrix metalloproteinase 13 (*mmp13*) (Figure 6). The *Vibrio* extract elicited a significant increase in expression of *il1b*, *vig1/rsad2*, *mx3*, *il10* and *mmp13*, while poly I:C elicited significant increases in expression of typical ISGs *vig1/rsad2*, *mx*, *il10* and *mmp13*. The significant increases in expression compared to controls confirmed that stimulated fish were undergoing a strong immune reaction.

The 23 *irf* genes within salmonids displayed a wide range of diversity in their expression levels after stimulation with poly I:C or Vibrio extract (Figure 5B). More than half of the IRFs examined (14 out of 23) displayed significant changes following stimulation, indicating that a large majority are involved in salmonid immunity in some way. IRF family members: *irf1-2*, *irf2-2*, *irf3*, *irf5-1*, *irf6-1*, *irf7-1*, *irf7-2*, *irf9-1*, *irf9-2*, *irf10-2* and *irf11*-*2* were all significantly upregulated in expression following poly I:C stimulation whereas *irf4b1* and *irf4b2* displayed a significant decrease in their expression levels following poly I:C stimulation. After Vibrio stimulation, only *irf11-2*, *irf7-1* and *irf9-1* displayed a significant increase in expression, whereas *irf5-2* displayed a significant decrease in expression in *Vibrio*-stimulated fish.

## 4. Discussion

The IRFs are an ancient family of proteins central to the regulation of interferon activity. Despite the importance of this family, there are still gaps in the knowledge of the fish-specific IRFs related to evolution, nomenclature and function, which is then exacerbated within the salmonids due to the ssWGD. The aim of this work was to address the evolution of the IRF family within the salmonids and provide resources to analyse expression and functions for the salmonid IRFs (PCR primers, coherent nomenclature). The recent availability of well-assembled genomes for six salmonid species allowed a first exhaustive description of the *irf* gene repertoire.

We initially identified 23 IRF family members within 6 salmonid species which had no apparent consensus on nomenclature. Two complementary approaches of phylogeny and synteny were used to determine the sub-families each IRF belonged to and their evolutionary history in regard to the various whole-genome duplications in fish. This initial approach revealed that almost all of the salmonid IRFs were products from the ssWGD with an exception in regard to *irf4a/irf4b* which were likely a result of the tsWGD. While there is only one copy of *irf2* in most fish groups, we identified the presence of two *irf2* copies within zebrafish, catfish and herring, in addition to Salmonids. In fact, the two *irf2* copies found in salmonids have likely arisen from the ssWGD, independently from the second copy in the above species. An additional copy of *irf2* (named *irf2a*) has been generated in a few other fish groups. The *irf2a* genes found in catfish and zebrafish are true loco-orthologs. These two species belong to evolutionarily related groups, and comparative genomic studies have revealed that they show a high level of syntenic conservation [38,39]. Due to no further WGDs within zebrafish or catfish, these extra *irf2* genes may be assumed to have been retained from the tsWGD. In contrast, the second *irf2* copy found in herring was not in the same genomic context as in zebrafish and catfish. Thus, these different “*irf2a*” genes found in non-salmonids may have been produced either at the tsWGD, or independently by punctual gene duplication.

We also identified the presence of an 11th IRF member in fish species belonging to the IRF-1G evolutionary group. Huang et al. [24] first recognised this fish-specific member in their characterisation of the IRF family in vertebrates and named it *irf11*; however, the name *irf1a* was mostly used in many species. As noted in our previous work in zebrafish [40], we see here that *irf11* was found to be an outgroup to the *irf1* and *irf2* clades within the IRF-1G group. Further comparative synteny analysis of the *irf1* and *irf11* genes found no common gene content/order between the genomic neighbourhood of these two groups of genes. Interestingly, salmonid *irf11* shared almost identical gene order with the elephant shark, a cartilaginous species which diverged from bony fish 450 MYA [41]. While phylogenetic analysis shows that these genes form a well-supported group, examination of the other fish species genomic neighbourhoods would suggest that *irf11* was subjected to multiple rearrangements over the evolution of the various teleost groups. From the small subset of fish species analysed in our study, the rearrangement rate of *irf11* would appear to be quite high, as three distinct gene neighbourhoods can be seen to be conserved across: salmonids and elephant shark; zebrafish, catfish and herring; and stickleback and fugu.

Normally following a WGD event, the resultant genome eventually only retains a fraction of duplicated genes, as the redundant genes are gradually inactivated through a process known as “gene fractionation” [42]. In salmonids, this phenomenon is illustrated by the large percentage of duplicated genes still present within the genome (48% of genes with retained ohnologues) which is likely due to the relatively recent ssWGD (88–103 Myrs ago), compared to the genomes of other fish that retain much less pairs from the tsWGD (~300 Myrs ago) [28,43]. Salmonid *irf* paralogs resultant from the ssWGD identified within this paper appeared to be almost all conserved bar *irf3* (no duplicated copy could be located in any salmonid species) with no pseudogenes and a clear assignation to subgroups. Interestingly, this is in sharp contrast with the STAT family of transcription factors, in which the retention/loss rate is very variable across members [37]. While both STAT and IRF are key transcription factors of the IFN signalling, these contrasted evolutionary pathways suggest different dynamics of sub/neofunctionalization after the ssWGD [44,45]. Another possible factor in the selection pressure these families face is from viral subversion, where viruses have evolved strategies to avoid detection from the immune response [46]. IRFs 3 and 7 are common targets from several viruses and accessory proteins from paramyxoviruses have been shown to mimic IRF3 in order to phosphorylate with TBK1 leading to its degradation instead of the induction of the antiviral response [47]. Although this has been studied in mammals, it may be no coincidence that IRF3 is commonly targeted due to the lack of a second paralog that could confer resistance.

While induction of a paralog by poly IC or bacterial extracts suggests it is somewhat involved in the immune reaction, the lack of modulation does not imply a lack of a key role. Thus, our recent KO experiment of *stat1a* gene, which was not inducible by type I IFN or viral infection, established that it is required for a typical induction of ISG [48]. Hence, we are aware that further studies are necessary to evaluate the role of each paralog in the IFN response. Depending on a whole host of factors, different IRFs are noted to be positive or negative regulators (or even both) of the human/mouse interferon response, however the major consensus is IRF1, IRF3, IRF5, IRF7 and IRF9 function as positive mediators of the type I interferon response while IRF2 and IRF4 act as regulators [7,49,50]. The large repertoire of *irf* paralogs in salmonids offer a unique opportunity to test how these functions are conserved or have quickly diversified.

The profile of up- and down-regulation of *irf* by following an in vivo challenge with *V. anguillarum* extract or poly I:C suggests that most members of the IRF family are involved in the immune reaction in salmonids. Within our results it can be seen that over half of the salmonid IRFs (*irf11-2*, *irf1-2*, *irf2-2*, *irf3*, *irf4b1*, *irf4b2*, *irf5-1*, *irf5-1*, *irf6-1*, *irf7-1*, *irf7-2*, *irf9-1 irf9-2 and irf10-2*) are modulated in some way in response to bacterial or viral stimulation, while only two subfamily’s *irf* paralogues showed no statistically significant changes due to stimulation: *irf4a* and *irf8*. In mammals, IRF3 and IRF7 are directly involved in the transcriptional induction of type I IFN-α/β genes following their activation while IRF1, IRF5 and IRF9 (part of the ISGF3 complex with STAT) are involved with positive regulation of the IFN response and stimulation of ISGs [51,52,53,54,55]. In our study, at least one paralogue from each salmonid homolog of these genes was induced following poly I:C stimulation. Salmonid *irf* homologs of the mammalian genes involved in regulation of the IFN response (*irf2* and *irf4*) [56,57] showed an increased (*irf2-2*) or a decreased (*irf4b1* and *irf4b2*) expression in poly I:C-stimulated fish. Modulation of these genes would suggest that at least one paralog from each of these IRF sub-families was involved in the viral immune response like their mammalian counterparts which have key roles in IFN signalling. Similarities between both mammalians and salmonids can be seen in the large repertoire of type I IFN genes in both classes [16,58] which then converges upon their interaction with a relatively small number of IFN receptors. Mammalian type I IFN signalling occurs through the IFNAR1/R2 receptor while fish signal through homologues of type I IFN receptors made of CRFB1/2 and CRFB5, of which salmonids possess several paralogues that their various type I IFNs subtypes can signal through [17,59,60,61]. This difference in the number of retained *irf* genes and IFN receptors in fish (and especially in salmonids) compared to mammals would indicate regulation of the IFN response likely differs, offering a large set of possibility for *irf* sub-functionalization.

The difference in knowledge of IRF function in fish is further exemplified by the additional IRFs 11 and 10 whose function is not well understood due to their absence in mammals. The salmonid-specific *irf11-2* within our study was strongly induced following poly I:C stimulation, with the largest fold change of all the IRFs examined and the lowest constitutive expression in the unstimulated control fish. This low constitutive expression of IRF11 in relation to the other IRFs has also been observed in mandarin fish (*Siniperca chuatsi*) [62]. In zebrafish, the unique IRF11 has been shown to restrict viral reproduction through the induction of IFN and ISGs indicating it as a positive mediator of IFN [63]. Taken together, these observations infer salmonid *irf11-2* as likely playing some role in activating the IFN response similar to zebrafish. However, there are various contradicting reports on IRF11 expression in various fish species: poly I:C but not actual viral infection increased expression of IRF11 in some tissues of mandarin fish [62]. Poly I:C, LPS, *V. anguillarum*, and *S. aureus* all caused a significant decrease in IRF11 expression in spleen tissue of miiuy croaker 24 h following stimulation, although there were increases observed in some tissues 6 h after [64]. In addition, a significant contribution of the non-induced salmonid *irf11-1/irf1a-1* is also possible but was not seen in our expression data. IRF10 has been shown to have a regulatory role in the induction of IFN and ISGs. Over-expression of IRF10 in common carp induced a downregulation of ISGs after poly I:C stimulation while in zebrafish over-expression of IRF10 blocked the induction of type I IFNs IFN1 and IFN3 [65,66]. A previous characterisation of the two *irf10* paralogues in rainbow trout revealed that *irf10-2* may be more important in the antiviral response which is in agreement with our results showing only one *irf10* paralogue was significantly modulated [27].

## 5. Conclusions

The high stability of the IRF repertoire in salmonids calls for a detailed functional characterization, which will require loss and gain of function experiments. The data presented here will certainly contribute to disentangling the remarkable complexity of the IFN system in this fish group. Interestingly, several of the paralogs (i.e., *irf5*, *irf7*, *irf9*) characterised here have shown differential responses to viral or bacterial stimulation which could indicate the evolution of new phenotypes within these genes, however the answers to this require future research and the use of gene knockout.

## Figures and Tables

**Table 1:** Rainbow trout primer sequences used for qPCR with gene IDs and NCBI accession numbers.

Gene ID	Gene Name	Direction	Sequence	Annealing	Product Size (bps)	Accession
100135845	β actin ^1^	Forward	GGTGGTAGGCCAGAGGC	60	101	NM_001124235.1
		Reverse	GGGAGAAGATGACCCAGATCATG			
100136024	IL1b ^2^	Forward	GGAGAGGTTAAAGGGTGGCGA	60	121	XM_036979104
		Reverse	TGCCGACTCCAACTCCAACA			
110494493	MX3	Forward	CCTCCTGAAATCAGCGAAGAC	60	364	XM_021569609.2
		Reverse	GAGTCTGAAGCATCTCCCTCCTG			
100136835	IL10 ^2^	Forward	CGACTTTAAATCTCCCATCGAC	60	70	NM_001245099.1
		Reverse	GCATTGGACGATCTCTTTCTTC			
100135876	VIG1 ^3^	Forward	GGCAACTCCAAGCAGTGTCAA	60	187	XM_021582972.2
		Reverse	GTCGTGTATGAAAGGCTCTCCG			
100136064	TNFa2 ^2^	Forward	GGAGGCTGTGTGGCGTTCT	60	73	NM_001124374.1
		Reverse	TGCTGACACCAGGCAAAGAG			
100136017	MMP13 ^2^	Forward	GCACCTTCTCTCTGCCCCGC	60	235	XM_021618131.2
		Reverse	AGGCTCTGTTGTGGTTTGCTGC			
110499402	IRF11-1 (a1)	Forward	TTGATGAGACAGCTCAAGTTTTC	55	146	XM_021576528.1
		Reverse	CTTAGGATCAGGTTCGTCTTTC			
110502724	IRF11-2 (a2)	Forward	CCAGGGGTCACCTGGCG	62	169	XM_021580977.1
		Reverse	TCCATGTCTTCGGATCAGGC			
110533376	IRF1-1	Forward	TTACAAAATGCTGAGCGTCAG	55	237	XM_021617512.1
		Reverse	GTCTCCCCTACGTTGTCTGA			
100135950	IRF1-2	Forward	GATGAAGAACGTCCACTCC	55	231	NM_001124293.1
		Reverse	AAATCATCTAGGCTGTCTGT			
110519953	IRF2-1	Forward	ATGCGAATGCGACCATGGC	55	195	XM_021596860.1
		Reverse	GTATGAATGGCCCAGTTCTTG			
100136151	IRF2-2	Forward	TGGAACAGATAAACTCTTC	62	130	NM_001124438.1
		Reverse	ATAAATAAAGGAGCGTCTTTC			
100750229	IRF3	Forward	AGCAATGGTAGGGTTCAAGG	60	179	NM_001257262.1
		Reverse	CATCTGGCCACTGGAACAG			
100499175	IRF4-a1	Forward	CCCACATGAGCTCAGTCAATAG	60	139	XM_021613502.1
		Reverse	GGGTCGGCTGAGTGGCTG			
100499174	IRF4-a2	Forward	CGATCAGATTAACAGCAGTAG	60	130	NM_001310139.1
		Reverse	CATCCTCCTCTCGATTGTAG			
110521762	IRF4-b1	Forward	GCTCGTGCAGCGAAGTCAG	60	181	XM_021599601.1
		Reverse	AGGCATCTGTGTCTGCAGG			
110524663	IRF4-b2	Forward	TCCGGATTCGGACTACGGC	60	110	XM_021604510.1
		Reverse	TCTCCCACACGAGGCCTGC			
110500261	IRF5-1	Forward	AGCATTACCATGGCAGCGC	60	130	XM_021577521.1
		Reverse	TGTTGGAGGGTCCTACCG			
110500261	IRF5-2	Forward	AGCATTACCATGGCAGCGC	60	130	XM_021577521.1
		Reverse	TGTTGGAGGGTCCTACCG			
110528098	IRF6-1	Forward	GGATGAAGATGAATCAGATGGC	60	209	XM_021609915.1
		Reverse	GGGACGAAGGCTGCATCTC			
110494340	IRF6-2	Forward	AGACAACAAGCGCTTCAGGG	60	111	XM_021569306.1
		Reverse	TGGAACTTTCCTGTCTCCAC			
100750228	IRF7-1 (a)	Forward	AGCAATACACTGGTTTGTTC	60	145	XM_021600499
		Reverse	GTGGGATGCTCATTGATTTTC			
110497044	IRF7-2 (b)	Forward	GCCGGGTTGTGTTTTGTG	60	144	XM_021573049.1
		Reverse	CTTGTCATTGGGATGCGTG			
110526480	IRF8-1	Forward	TGGGAGGACGACAGTCGCAC	60	95	XM_021607480.1
		Reverse	GCCTTGAAGATAGAGGCGTCG			
110506608	IRF8-2	Forward	GTCTGGGAGGACGACAGC	60	98	XM_021586344.1
		Reverse	GCCTTGAAGATAGAAGCGTCT			
110535315	IRF9-1	Forward	TCCGATGGGGGTCGTGTG	60	160	XM_021620234.1
		Reverse	CCAACACTTGTTCATTCATC			
110489699	IRF9-2	Forward	TGTCTGAGGGGTGTCATGC	60	146	XM_021562471
		Reverse	GATGGGTACGAGGCGGTAG			
110492403	IRF10-1 (a)	Forward	CTTACCTGGGAGAACGAAG	60	151	XM_021566691.1
		Reverse	GACGAGTCTTCCAGGTG			
110532004	IRF10-2 (b)	Forward	ATCTGAATGAAGATGCAGCC	60	172	XM_021615574.1
		Reverse	CGCTCTGGGACCTCCTG			

^1^ Ref [34], ^2^ Ref [35], ^3^ Ref [36].

## Data Availability

All data reported in this work are shown in Figures, Tables and Supplementary Material.

## Supplementary Materials

The following are available online at https://www.mdpi.com/2073-4425/12/2/238/s1, Table S1. Full repertoire of Salmonid *irf* genes. Table S2. Genomic Neighbourhood of all Salmonid *irf* genes. Figure S1 Maximum likelihood phylogenetic tree of all IRF. Figure S2 Maximum likelihood phylogenetic tree of IRF1 and IRF2.
