# Supplementary material for: Evolution of the IRF Family in Salmonids
Source: Genes (Basel). 2021 Feb 8;12(2):238. doi: 10.3390/genes12020238 (PMC7915476; doi:10.3390/genes12020238)
Supplement: Supplementary file 1 [file genes-12-00238-s001.zip › supp data/FigureS1_revised.pdf]

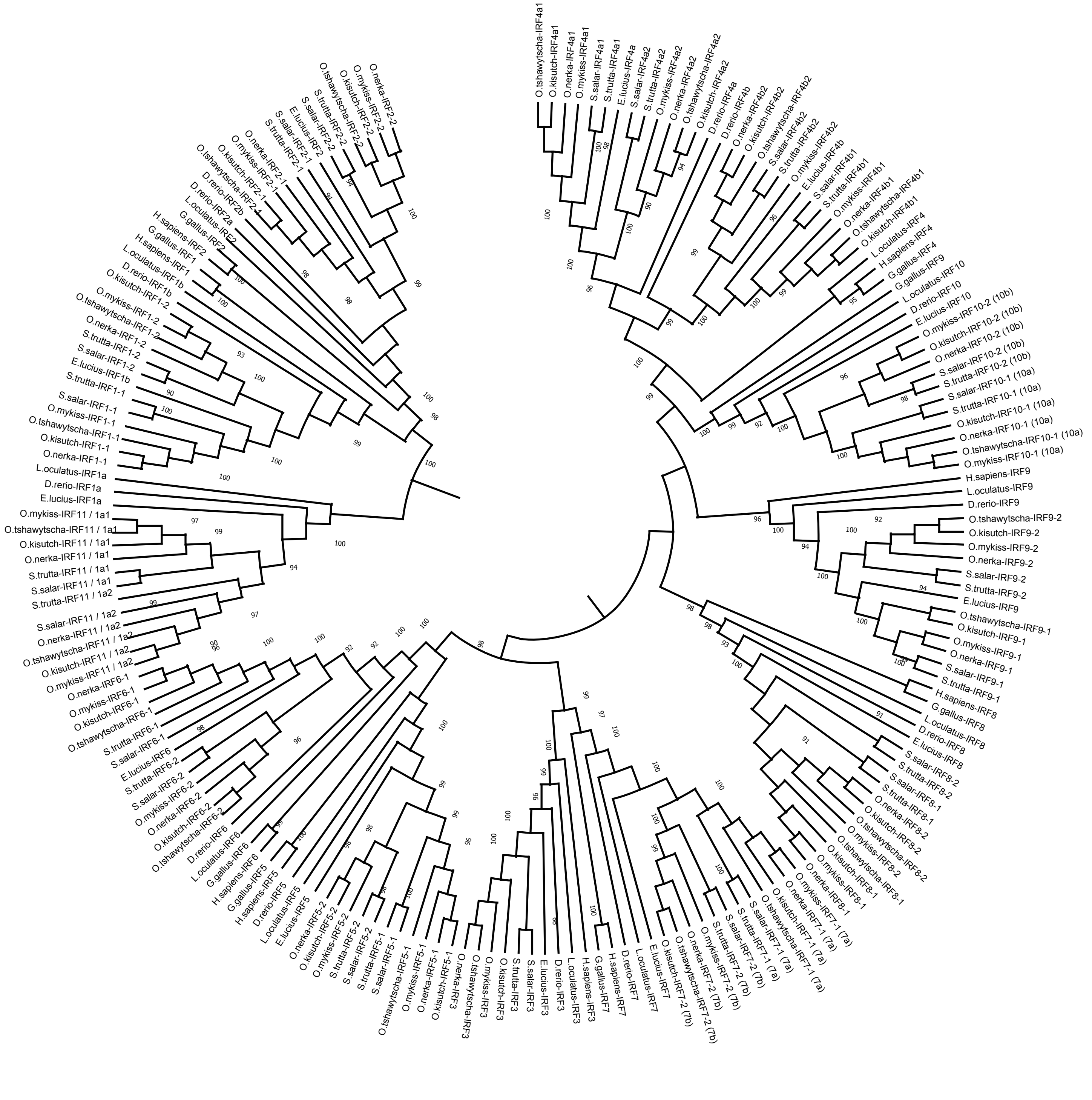

Figure S1. Phylogenetic tree showing the evolutionary relationship of IRF transcription factors in salmon -ids. IRF protein sequences were aligned using ClustalW in the MEGA-X software. Following alignment, the phylogenetic tree was constructed using the maximum-likelihood method in MEGA X and corrected using the poisson model. The branch support values were gained by non-parametric bootstrapping (500 replicates). The scale bar represents the calculated evolutionary distance. Genbank accession numbers for all species can be viewed in Table S1.
