# Supplementary material for: Evolution of the IRF Family in Salmonids
Source: Genes (Basel). 2021 Feb 8;12(2):238. doi: 10.3390/genes12020238 (PMC7915476; doi:10.3390/genes12020238)
Supplement: Supplementary file 1 [file genes-12-00238-s001.zip › supp data/Figure S2 revised.pdf]

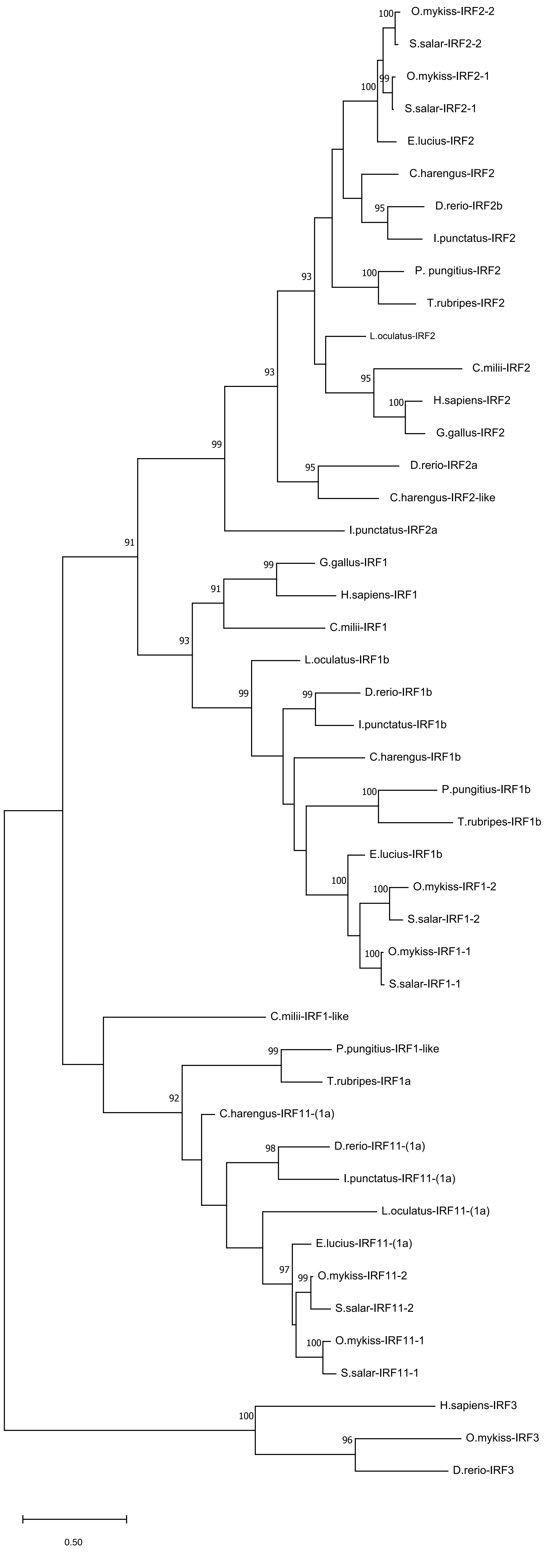

Figure S2. Phylogenetic tree showing the evolutionary relationship of IRF1 and IRF2 transcription factors in vertebrates with IRF3 used as an outgroup. IRF protein sequences were aligned using ClustalW in the MEGA X software. Following alignment, the phylogenetic tree was constructed using the maximum-likelihood method in MEGA X and corrected using the poisson model. The branch support values were gained by non-parametric bootstrapping (500 replicates). The scale bar represents the calculated evolutionary distance. Genbank accession numbers for all species can be viewed in Table S1.
